# DEMENTIA CARE THROUGHOUT THE TRAJECTORY AND ACROSS SETTINGS: RESULTS FROM THE DEVELOP AD RESEARCH STUDY

**DOI:** 10.1093/geroni/igac059.1623

**Published:** 2022-12-20

**Authors:** Lauren Hunt, Katherine Ornstein, Lauren Massimo

## Abstract

About 1 in 8 older adults in the United States has dementia, making it one of the biggest public health issues facing society today. The impact of dementia ranges across health and social systems, yet consideration of these contexts--which are of great relevance to patients and families--has been underexplored in prior research. In this symposium, we will present findings from the Deploying High-Value Longitudinal Population-Based data in Dementia Research (DEVELOP AD Research) study. This multicenter collaboration leverages existing population-based data sources to examine the health and social impacts of dementia throughout the trajectory of decline and across acute, long-term, and community-based settings. Session one will describe how acute care utilization increases in the years before the onset of incident dementia. Session two will examine the association between dementia and increased mortality risk after disruptive medical events, including hip fracture and pneumonia. Session three will report on the prevalence and patterns of medication overuse and misuse in community-dwelling people with dementia. Session 4 will estimate how many community-dwelling individuals, including those with dementia, could benefit from home-based medical care. Session 5 will focus on hospice use patterns and hospice care quality rating in people admitted to hospice for dementia versus other conditions. Together, these presentations provide a wide lens on the health and social experiences and needs of people with dementia at the national level, leading to a better understanding of how society can better address the challenges of caring for this growing population.

